# MiR-204 silencing in intraepithelial to invasive cutaneous squamous cell carcinoma progression

**DOI:** 10.1186/s12943-016-0537-z

**Published:** 2016-07-25

**Authors:** Agustí Toll, Rocío Salgado, Blanca Espinet, Angel Díaz-Lagares, Eugenia Hernández-Ruiz, Evelyn Andrades, Juan Sandoval, Manel Esteller, Ramón M Pujol, Inmaculada Hernández-Muñoz

**Affiliations:** 1 Department of Dermatology, Hospital del Mar, Universitat Autònoma de Barcelona, Barcelona, Spain; 2 Group of Inflammatory and Neoplastic Dermatological Diseases, IMIM (Hospital del Mar Medical Research Institute), Barcelona, Spain; 3 Cytogenetics Molecular Biology Laboratory, Department of Pathology, Hospital del Mar, Universitat Autònoma de Barcelona, Barcelona, Spain; 4 Cancer Epigenetics and Biology Program (PEBC), Bellvitge Biomedical Research Institute (IDIBELL), L’Hospitalet, Barcelona, Spain; 5 Department of Dermatology, Hospital Universitari Vall d’Hebron, Barcelona, Spain; 6 Cancer Epigenetics and Biology Program (PEBC), Bellvitge Biomedical Research Institute (IDIBELL), Barcelona, Catalonia Spain; 7 Department of Physiological Sciences II, School of Medicine, University of Barcelona, Barcelona, Catalonia Spain; 8 Institució Catalana de Recerca i Estudis Avançats (ICREA), Barcelona, Catalonia Spain

**Keywords:** miR-204, Cutaneous squamous cell carcinoma, Actinic keratosis, Sun-induced keratinocyte intraepithelial neoplasia, DNA methylation, MAPK, STAT3

## Abstract

**Background:**

Cutaneous squamous cell carcinoma (cSCC) is the second most common skin cancer and frequently progresses from an actinic keratosis (AK), a sun-induced keratinocyte intraepithelial neoplasia (KIN). Epigenetic mechanisms involved in the phenomenon of progression from AK to cSCC remain to be elicited.

**Methods:**

Expression of microRNAs in sun-exposed skin, AK and cSCC was analysed by Agilent microarrays. DNA methylation of miR-204 promoter was determined by bisulphite treatment and pyrosequencing. Identification of miR-204 targets and pathways was accomplished in HaCat cells. Immunofluorescence and immunohistochemistry were used to analyze STAT3 activation and PTPN11 expression in human biopsies.

**Results:**

cSCCs display a marked downregulation of miR-204 expression when compared to AK. DNA methylation of miR-204 promoter was identified as one of the repressive mechanisms that accounts for miR-204 silencing in cSCC. In HaCaT cells miR-204 inhibits STAT3 and favours the MAPK signaling pathway, likely acting through PTPN11, a nuclear tyrosine phosphatase that is a direct miR-204 target. In non-peritumoral AK lesions, activated STAT3, as detected by pY705-STAT3 immunofluorescence, is retained in the membrane and cytoplasm compartments, whereas AK lesions adjacent to cSCCs display activated STAT3 in the nuclei.

**Conclusions:**

Our data suggest that miR-204 may act as a “rheostat” that controls the signalling towards the MAPK pathway or the STAT3 pathway in the progression from AK to cSCC.

**Electronic supplementary material:**

The online version of this article (doi:10.1186/s12943-016-0537-z) contains supplementary material, which is available to authorized users.

## Background

Cutaneous squamous cell carcinoma (cSCC) is an epidermal keratinocyte derived skin malignant tumor with an incidence of 16 per 100,000 people in Europe [1]. Chronic solar radiation exposure is the most important risk factor for cSCC development since cSCC preferentially arises from precancerous lesions such as UV-induced actinic keratosis (AK) [2]. AK is a keratinocyte intraepithelial neoplasm (KIN or in situ cSCC) that may progress to invasive cSCC at a rate estimated between 0.025 and 16 % for an individual lesion per year [3], and patients with multiple AKs have an annual risk of developing invasive cSCC ranging from 0.15 to 80 % [4]. While most cSCCs are cured by surgery and/or radiotherapy, a subset of tumors may show an aggressive clinical behaviour, with increased local recurrences and metastases [5]. Patients with metastatic cSCC have a disease-specific survival at 1 year of 44–56 % [6].

The genetic basis for the progression from actinic keratosis to cSCC is not completely understood [4, 7–10]. TP53 mutations are an early phenomenon already seen in 40 % of in situ cSCC [11]. *NOTCH* inactivating mutations have recently been found in ~75 % of cSCC [12] but information about their presence in AK is lacking. Although increased genomic instability is found in cSCC, AKs already harbour cytogenetic alterations at several loci [13]. We previously demonstrated that *EGFR* and *MYC* gains are already frequent in AKs and not significantly increased in invasive cSCC [14, 15]. Thus, further genomic and epigenetic studies are needed to better understand the progression from AK to cSCC.

MicroRNAs (miRNAs) are a class of small non-coding RNAs that can regulate gene expression of a very broad set of targets including loci in the protein-coding region of mRNAs, 5′ UTRs, intronic and intergenic transcripts and other non–protein-coding RNAs [16]. MiRNAs are proposed to regulate 60 % of all protein-coding genes in humans [17]. Every miRNA can have multifunctional roles as either repressors or enhancers of their targets expression by diverse pathways [18].

Aberrant miRNA expression is linked to cancer development and progression and affects several processes including proliferation, apoptosis, differentiation and invasiveness. In cSCC most of the altered miRNAs are downregulated (miR-125b, miR-34a, miR-214, miR-124, miR-361, miR-193b, miR-365a, miR-20a, miR-199a) [19–25] whereas only a small number of miRNAs have been found to be upregulated and act as onco-miRNAs, being involved in angiogenesis, colony formation, migration and invasion, and metastatic spread (miR-365, miR-9, miR-184, miR-21, miR-31, miR-135b, miR-205, let-7a) [25–34]. However, most of these miRNAs have been identified by comparing miRNA expression in cSCC versus healthy skin, and only few studies were aimed to compare miRNA expression in AKs and cSCC [19, 23, 31]. Here we explore the miRNA expression signature of cSCC and AK to identify miRNAs implicated in the development and evolution from AK to cSCC.

## Methods

### Clinical samples

All samples were obtained at the Department of Dermatology of Hospital del Mar (Barcelona, Spain) and immediately embedded in OCT and frozen in liquid nitrogen.

### Microarray analysis

Four millimeter punch biopsies were taken from moderately differentiated cSCCs (20 lesions in sun-exposed areas from 20 patients). Five KIN3 actinic keratoses and 5 control chronically sun-exposed (CSE) skin samples were obtained by shave biopsy from 10 different patients. Clinicopathological data of the patients can be found in Additional file 1: Figure S1. Samples were macrodissected from frozen blocks after examination of hematoxylin-eosin stained sections such that all samples displayed a minimum 70 % enrichment for tumor or dysplastic cells. RNA quality and concentration was measured using the Agilent Bioanalyser. Only samples with RIN ≥6 (Bioanalyzer 2100, Agilent Technologies) were included in the study. RNA (10 ng) was used as template for whole transcriptome amplification and cDNA synthesis. The microarray technology used was Agilent Human miRNA microarrays (V2, G4470B, Agilent Technologies, Sanger Database version 10.1). Data normalization was performed with Feature Extraction Software (Agilent Technologies). Extracted intensities were background corrected using the *normexp* method with an offset of 50 [35]. Background corrected log2-transformed intensities were normalized using invariants normalization to make data from all arrays comparable [36]. Microarray probes were collapsed to miRNAs by taking the median intensity of the respective probes per miRNA. For determining differentially regulated miRNAs moderated t-tests were applied using Limma (Linear Models for Microarray Data) [37]. MiRNAs with FDR adjusted *p*-value <0.05 and additionally a fold change exceeding 1.2 in absolute value were selected as the relevant ones. All statistical analyses were performed with the Bioconductor project in the R statistical environment [38].

### DNA methylation analysis by pyrosequencing

Bisulphite conversion of 500 ng of each DNA sample was performed with EZ DNA Methylation-Gold Kit (Zymo Research, Milan Italy) according to the manufacturer’s recommendations. PCR for miR-204 promoters was performed with 1 μl of bisulphite converted DNA under standard conditions with biotinylated primers using an annealing temperature of 60 °C. Primer sequences are: miR-204 Forward (5′-[Btn]TGGTTTTTTTTTAAATTAAGTTAGTAAAGT-3′); miR-204 Reverse (5′- ACAACCTACACAAAACAACCTATAATC-3′); miR-204 Sequencing (5′-ACAACCTACTCCAAAAT-3′) and were designed with PyroMark Assay Design 2.0. PCR products were observed on 2 % agarose gels before pyrosequencing analysis. Reactions were performed in a PyroMark Q96 System version 2.0.6 (Qiagen, Milan, Italy) and the methylation values of the CpG dinucleotides were obtained using Pyro Q-CpG 1.0.9 (Qiagen, Milan, Italy).

### Cell culture and expression arrays

HaCaT cells were cultured in DMEM medium (Sigma) supplemented with 10 % fetal bovine serum in standard conditions. SCC13 and SCC12 cells were cultured in DMEM:F12 medium (Sigma) supplemented with 10 % fetal bovine serum, penicillin–streptomycin (100 mg/ml, Invitrogen), adenine (180 mM, Sigma), human recombinant EGF (10 ng/ml Peprotech), insulin (5 mg/ml, Sigma), cholera toxin (0.1 nM, Gentaur) and hydrocortisone (0.5 mg/ml, Sigma). Human epidermal keratinocytes (HEK, Life Technologies) were cultured in KSFM medium (Thermo Fisher). To restore the expression of DNA-methylated miR-204, HaCaT cells were treated with the DNA demethylating agent 5-aza-2′-deoxycytidine (Sigma) at 10 μM for 120 h. To generate retroviral stocks for stable expression of miR-204 antagomir, 293T cells were transfected with Fugene (Roche) with miRZIP-204 anti-miR-204 construct (shRNA targeting miR-204 microRNA, cloned in pGreenPuro HIV based lentivector, BioCat), or with the corresponding pGreenPuro scrambled hairpin negative control (BioCat). HaCaT cells were transduced with lentiviral supernatants in the presence of Polybrene (4 μg/ml; Sigma). Two days after the infection, control and miR-204 antagomir expressing HaCaT cells were FACS-sorted. Total RNA extraction from GFP-positive cells was performed with Genelute mammalian total RNA kit (Sigma). Samples were then processed following the protocols provided by Ambion (Ambion WT Expression, Protocol Number 4425209; Revised B 05/2009) and Affymetrix (Affymetrix WT Terminal Labeling and Hybridization User Manual, Protocol Number 702808 and Expression, Wash, Stain and Scan User Manual Protocol Number 702731). The expression platform used was Human Gene 2.0 ST (Affymetrix®).

Transient expression of miRIDIAN microRNA human hsa-miR-204-5p-hairpin inhibitor (Dharmacon), pre-miR™ miRNA-204-5p precursor (Life Technologies) or the corresponding controls (miRIDIAN microRNA hairpin inhibitor negative control #1, Dharmacon, and pre-miR™ miRNA precursor negative control #1, Life Technologies, respectively) at 50 nM was performed with DHARMAFECT 4 as described earlier [39]. Twenty four hours later, cells were treated with 10 ng/ml EGF, IL6 or FGF9 (Peprotech) for the times indicated, in the absence or the presence of 50 μM NSC87877 (Sigma).

### RNA extraction and quantitative reverse transcription PCR (qRT-PCR)

RNA was extracted from frozen biopsies with the mirVana™ miRNA Isolation Kit (Applied Biosystems). qRT-PCR for miR-204 transcripts was performed with TaqMan® microRNA assays (Applied Biosystems), using RNU48 levels to normalize the data. Real-time PCR reactions were performed in triplicate in 384-well plates and were run in a QuantStudio™ 12K Flex Real-Time PCR System. The 2-(ΔΔCt) method [40] was used to determine relative quantitative levels of miR-204. qRT-PCR to analyze gene expression was performed using SYBR Green PCR master mix (Applied Biosystems, Life Technologies) and GAPDH expression was used as endogenous control for normalization purposes. Primers used for qRT-PCR were: PTPN11 (5′- AAAGGGGAGAGCAATGACGG-3′; 5′- GGGGCTGCTTGAGTTGTAGT-3′); MFAP3 (5′-CCCGCTGTGCTTTTGTTGTA-3′; 5′-ATCCGAGTGGGAACTTGCTG-3′); FGF9 (5′-GCAGTCACGGACTTGGATCA-3′; 5′-TCCAGAATGCCAAATCGGCT-3′); FGF2 (5′- CCCCAGAAAACCCGAGCGA-3′; 5′- CGTCCGCTAATCTGGCACC-3′); CBL (5′- ATTCTCCATGGCCCCACAAG-3′; 5′- GAATATGGCCGGTCTGGAGG-3′); MAP3K2 (5′- GACCAACTGGAGATTGGGCA-3′; 5′- GGTCTCAGGACTATCGGGGT-3′); C1QBP (5′- TTTGCATCTGCACGTGTTCG-3′; 5′- CAGGAGCTGCCGGAAAGG-3′); PDP2 (5′- CTTGCAAACACGCCTACTGT-3′; 5′- GCACAAAAAGCTGCACCAGT-3′); IL6 (5′-AGCCACTCACCTCTTCAGAAC-3′; 5′-GTGCCTCTTTGCTGCTTTCAG-3′); GAPDH (5′-AGTCAGCCGCATCTTCTTTTG-3′; 5′-AAATCCGTTGACTCCGACCTT-3′).

### Protein extraction and Western blot

These protocols were performed following standard techniques. STAT3 (C-20), ERK2 (C-14) and PTPN11 (SH-PTP2 B1) antibodies were purchased from Santa Cruz Biotech. Activated MAP kinase (diphosphorylated ERK1/2) antibody was from Sigma. Lamin B1, Actin, phospho-Y705-STAT3 and phospho-S727-STAT3 antibodies were from Abcam. HRP-conjugated secondary antibodies were purchased from Dako. Western blot quantification was performed using Image J densitometry software. Intensity of individual bands was normalized to Actin signal and referred to control conditions.

### Transfection and luciferase assay for PTPN11 3′ UTR construct

Oligonucleotides corresponding to the PTPN11 3′UTR region containing the miR-204 binding site (5′-TCGAGTTGCAATTGTCAAGTGTTTTGTTGTAGCTTAGTATCCATAAGGGAAAC TTAGACTATAGACATAGATCTGC-3′; 5′-GGCCGCAGATCTATGTCTATAGTCTAAGTT TCCCTTATGGATACTAAGCTACAACAAAACACTTGACAATTGCAAC-3′) were annealed and inserted in the psiCHECK2 plasmid (Promega), immediately downstream from the stop codon of Renilla luciferase, and the sequence was confirmed by sequencing. For the luciferase assay, the transfection was performed using Fugene HD (Roche). Once ready, HaCaT cells were transfected with the reporter plasmid along with miR-204 antagomir, miR-204 mimic or with their corresponding hairpin inhibitor or precursor negative controls at 50 nM. Renilla and Firefly luciferase activities were measured consecutively using a dual-luciferase assay (Promega) 24 h after transfection. Renilla luciferase activity was normalized to Firefly luciferase activity expressed from the psiCHECK2 plasmid.

### Immunofluorescence and immunohistochemistry

Immunofluorescence was performed on 4 μm-thick tissue sections of 14 non-peritumoral AKs, 11 cSCC and their corresponding adjacent AK samples. Heat-induced antigen retrieval was performed in 10 mM citrate (pH 6) for 15 min in a pressure cooker. The slides were then incubated with STAT3 (phospho-Y705) antibody (Abcam) for 12 h. After washing, the Envision + antibody reagent was applied (Dako). Reactions were developed with the TSA™ Plus Cyanine3/Fluorescein System (PerkinElmer). Nuclei were counterstained with Hoesch and examined with a fluorescence microscope (Olympus BX61). Cases where pY705-STAT3 signal was detected in more than 20 % of cells were considered as positive. PTPN11 immunohistochemistry was performed by antigenic retrieval in 10 mM citrate (pH 6) for 15 min in a pressure cooker. After primary antibody incubation, the Envision + System-HRP antibody reagent was applied (Dako) and reactions were developed using diaminobenzidine (DAB, Dako).

## Results

### Identification of miRNAs differentially expressed in actinic keratosis (AK) and cutaneous squamous cell carcinoma (cSCC)

**Fig. 1:**
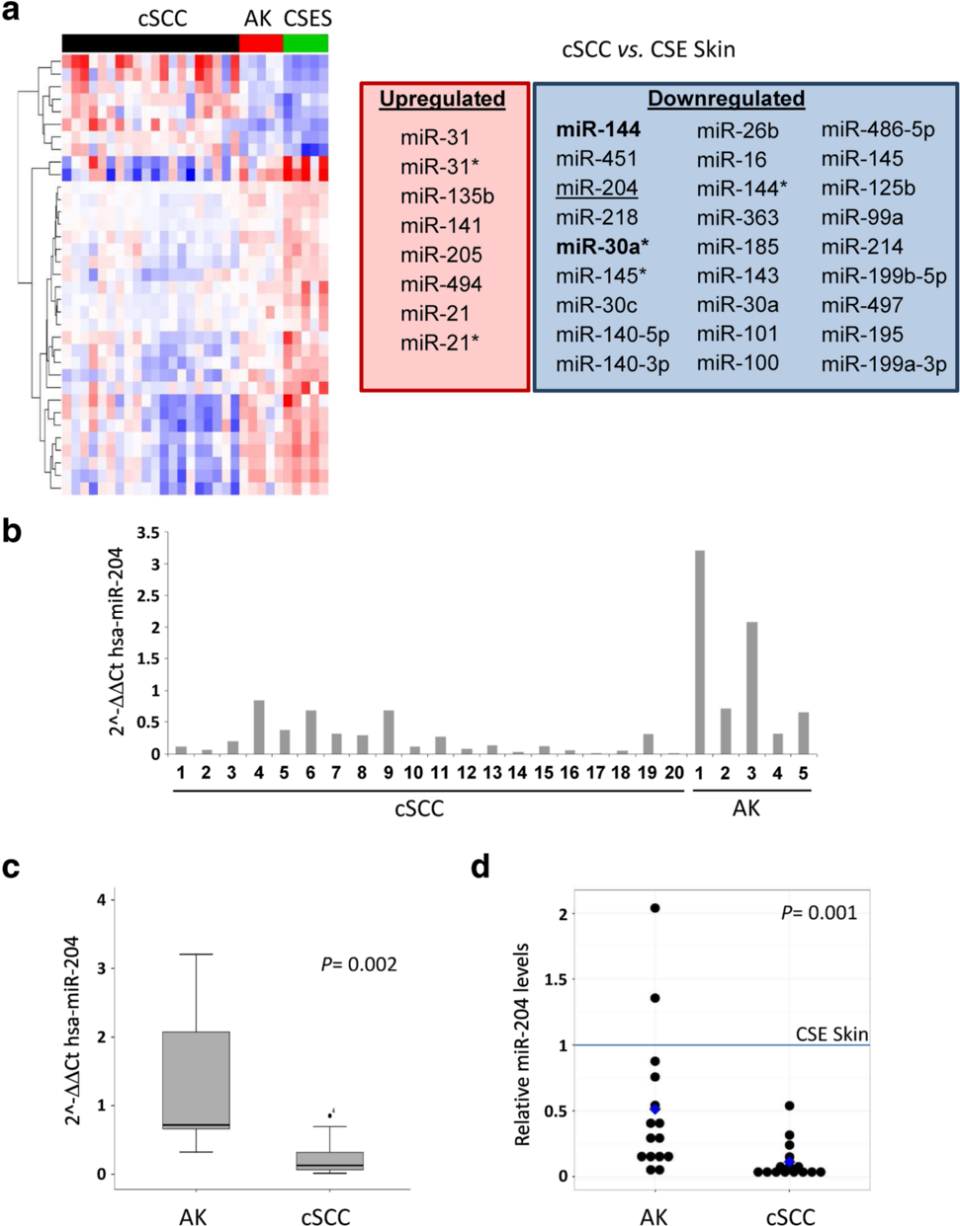
MiR-204 is differentially expressed in AK versus cSCC. **a** Supervised clustering of cSCC, AK and chronically sun exposed skin (CSES) expression arrays (*left*) and identification of differentially expressed miRNAs (*right*). **b** MiR-204 expression in cSCC and AK, analyzed by qRT-PCR and expressed as relative quantitative levels (2-(ΔΔCt) method). **c** *Box plot* representation of relative miR-204 levels in AK and cSCC. U-Mann Whitney test was applied to determine statistical significance. **d** Validation of miR-204 differential expression in an independent cohort. *Dot plot* representation of relative miR-204 levels in CSE skin, AK and cSCC from the same donor (*n* = 15), and referred to their corresponding miR-204 levels in CSE skin. Wilcoxon test was applied to determine statistical significance

To determine whether any miRNAs was aberrantly expressed in cSCC and in AK, 20 cSCC, 5 AK and 5 chronically sun-exposed (CSE) skin biopsy specimens were studied for miRNAs expression with a gene expression microarray (clinicopathological data, Additional file 1: Figure S1). Supervised clustering of cSCC vs. CSE skin expression arrays identified 35 miRNAs differentially expressed (Fig. 1a). In contrast, supervised clustering of AK vs. CSE skin identified only two downregulated miRNAs, miR-30* and miR-144, whereas no miRNA was found to be upregulated in AK. Supervised clustering of cSCC vs. AK identified a unique downregulated miRNA, miR-204 (Logarithmic fold change = −1.2; *p* = 0.0097) in cSCC, whereas no miRNA was found to be upregulated. Analysis of miR-204 expression by qRT-PCR in the 20 cSCC and 5 AK biopsies used for the array confirmed low to undetectable levels of this miRNA in cSCC, whereas AK displayed statistically significant higher levels of miR-204 (Fig. 1b, c). Independent validation in a different cohort consisting of 45 biopsy samples (15 CSE skins, 15 cSCCs and 15 cSCC-distant AKs from 15 patients; all lesions were located on the head and were at least 1 cm and maximum 5 cm apart from each other) confirmed miR-204 downregulation in cSCC when compared to intraindividual AKs (Fig. 1d).

### MiR-204 expression is frequently silenced by DNA methylation in cSCC

**Fig. 2:**
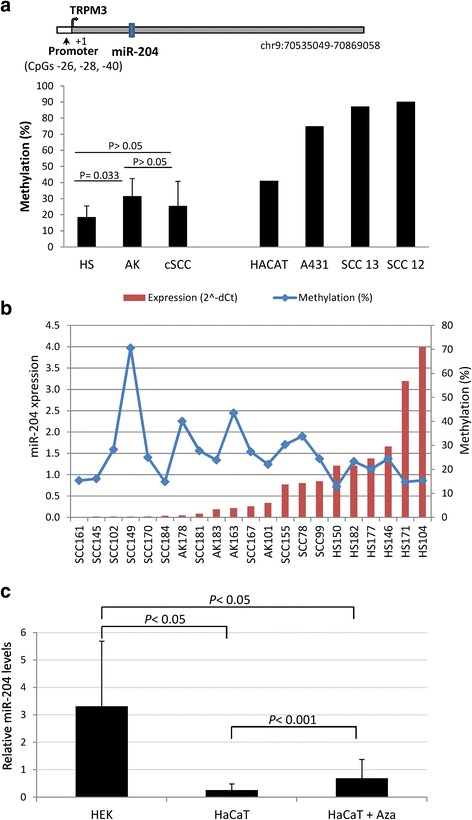
DNA methylation-associated silencing of miR-204 in cSCC cell lines and human samples. **a** *Up*, schematic representation of TRPM3/miR-204 gemomic locus. *Down*, DNA methylation levels of the miR-204 promoter region in cell lines and human biopsies. Methylation levels represent the mean of three consecutive CpGs (-26, -28, -40) located at the promoter region of miR-204. *Error bars* indicate standard deviation. **b** DNA methylation and expression levels of miR-204 in human biopsies detected by qRT-PCR. **c** Relative miR-204 levels in human epithelial keratinocytes and HaCaT cells, and restored expression of DNA methylated miR-204 in HaCaT cells after treatment with the DNA demethylating agent 5-aza-2′-deoxycytidine (Aza). Values were determined in triplicates by quantitative RT–PCR and are expressed as mean ± SD (*n* = 3)

MiR-204 is an intronic miRNA located at the TRPM3 gene that shares the same promoter and regulatory motif for transcription than TRPM3 [41, 42]. Pyrosequencing analysis of three consecutive CpGs (CpG -26, CpG -28, CpG -40) located at the miR-204 promoter region revealed that this promoter was heavily methylated in the two cSCC cell lines tested (SCC12 and SCC13) as well as in the epidermal carcinoma cell line A431, whereas the immortal keratinocyte cell line HaCaT displayed a partially methylated miR-204 promoter (Fig. 2a). Next we determined miR-204 promoter methylation in 5 CSE skin, 4 AK and 11 cSCC samples. Interestingly, an increase in miR-204 methylation levels was found from CSE skins to AKs, whereas DNA methylation only accounts for miR-204 silencing in seven of the cSCC samples (64 %) (cut off >24 %; the highest methylation value found in CSE skin biopsies) (Fig. 2a, b). Importantly, treatment of HaCaT cells, which express low but detectable miR-204 levels, with the demethylating agent 5-aza-2′-deoxycytidine partially restored miR-204 expression (Fig. 2c) suggesting that DNA methylation of the miR-204 promoter directly impinges on miR-204 expression. Altogether, these results indicated that different mechanisms account for miR-204 silencing, among them DNA methylation.

### Identification of miR-204 targets in non-transformed keratinocytes

**Fig. 3:**
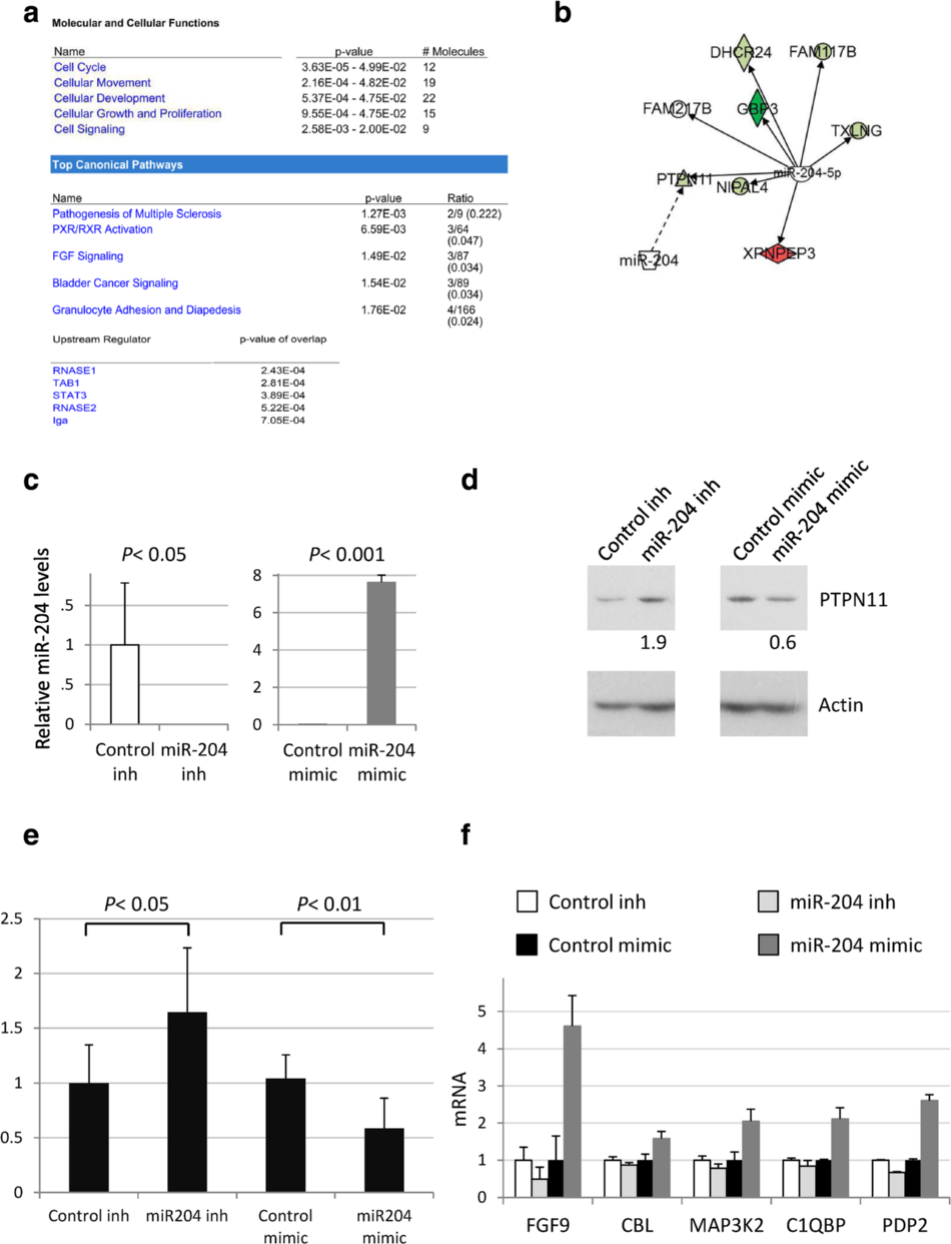
Identification of gene expression changes upon miR-204 downregulation in the non-tumorigenic HaCaT cell line. HaCaT cells infected with anti-miR-204 lentivirus (shRNA targeting miR-204) or with the corresponding pGreenPuro scrambled hairpin negative control lentiviral supernatant were flow cytometry-sorted and their total RNAs were purified. Differences in gene expression between both conditions were analyzed by the Human Gene ST Arrays platform (Affymetrix). **a** Ingenuity Pathway Analysis (IPA) identification of the molecular and cellular functions, top canonical pathways and upstream regulators. **b** In silico identification by IPA of putative direct miR-204 targets differentially regulated in HaCaT cells. **c** MiR-204 expression relative to RNU48 measured by qRT-PCR in HaCaT cells transfected with miR-204 antagomir or miR-204 mimic along with their appropriate controls. **d** PTPN11 expression is up-regulated by miR-204 in HaCaT cells, monitored by Western blot. **e** Relative Renilla luciferase activity derived from the PTPN11 3′ UTR reporter construct monitored after transfection of HaCaT cells with miR-204 antagomir or miR-204 mimic, along with their controls. **f** Independent validation of miR-204 regulation of some of the genes identified by the expression array, by HaCaT transient transfection of miR-204 antagomir or mimic along with their controls. Mean ± SD of three replicate samples from one representative experiment

Overall function of individual miRNAs in oncogenesis may be context dependent [43]. To determine gene expression changes induced by miR-204 downregulation in non-tumoral keratinocytes we infected HaCaT cells with lentiviral supernatants carrying short hairpin RNA (shRNA) targeting miR-204 or the corresponding scrambled hairpin negative control along with the GFP gene. Green-positive HaCaT cells were flow cytometry-sorted and their total RNAs were purified. Differences in gene expression between both conditions were analyzed by the Human Gene ST Arrays platform (Affymetrix) (GEO accession number: GSE71953 and Additional file 1: Figure S2). To identify molecular and cellular functions modulated by miR-204 in our cellular context we used Ingenuity Pathway Analysis (IPA) software. This analysis revealed that the expression array was significantly overrepresented by proteins that control cell cycle, cellular movement, cellular development, cellular growth, proliferation and cell signaling. Among the canonical pathways and upstream regulators, the FGF-STAT3 signaling pathway was identified to be deregulated in miR-204-depleted HaCaT cells (Fig. 3a). IPA software (which integrates experimentally demonstrated and predicted microRNA-mRNA interactions) identified a number of putative direct miR-204 targets (Fig. 3b). Among them, PTPN11 and FAM117B were also predicted to be miR-204 targets by the TargetScan 6.2 software [44].

PTPN11 (also called SHP2) is a member of the protein tyrosine phosphatase (PTP) family that directly affects STAT activity in A431 cell nuclei [45], while FAM117B is a protein-coding gene of unknown function expressed in the nucleus of basal keratinocytes [46]. Because the FGF-STAT3 pathway was identified to be deregulated in miR-204-depleted HaCaT we hypothesized that the PTPN11 phosphatase could be directly targeted by miR-204 to modulate STAT3 signaling. To investigate this possibility, HaCaT cells were transiently transfected with miR-204 antagomir (hairpin inhibitor), miR-204 mimic (pre-miRNA-204 precursor) or with the corresponding antagomir or precursor negative controls (Fig. 3c). According to the gene expression data, miR-204 antagomir transfected cells displayed lower PTPN11 mRNA levels than their control antagomir counterparts, whereas the expression of the miR-204 mimic enhanced PTPN11 levels (Additional file 1: Figure S3A). MiRNAs can silence cytoplasmic mRNA translation either by triggering an endonuclease cleavage, by promoting translation repression or by accelerating mRNA decapping [47]. Since miRNAs can interfere with mRNA translation in a manner independent of changes at the mRNA level [47] we tested miR-204 impact on PTPN11 protein expression. Contrary to the effect observed on PTPN11 mRNA levels, transfection of miR-204 antagomir in HaCaT cells up-regulated PTPN11 protein levels, whereas miR-204 mimic resulted in downregulation of PTPN11 expression (Fig. 3d). Next, we performed a reporter assay in which the Renilla luciferase reporter gene was under control of the PTPN11 3′ UTR. We observed that sequestration of endogenous miR-204 with the specific antagomir increased the expression of the Renilla reporter, while the expression of the miR-204 mimic reduced the expression of the reporter, confirming that PTPN11 may be a primary target of miR-204 through its 3′ UTR (Fig. 3e).

Finally we validated the microarray data by qRT-PCR analysis of some differentially expressed genes in HaCaT cells transfected with miR-204 antagomir or miR-204 mimic (Fig. 3f). The fact that miR-204 inhibition represses while miR-204 induces the expression of the validated genes suggests that miR-204 might indirectly regulate their expression. We also tested some cellular functions identified by IPA as modulated by miR-204 expression. Cell cycle status assessed by BrdU staining to detect DNA synthesis revealed that miR-204 depletion in HaCaT cells impairs BrdU incorporation while cells transfected with miR-204 mimic displayed an enhancement in BrdU staining (Additional file 1: Figure S3B). In contrast, cell migration enhancement was observed in HaCaT cells transfected with miR-204 mimic, whereas no effect was observed in those transfected with the antagomir (Additional file 1: Figure S3C). Therefore gene expression and migration modulation by miR-204 were more effectively observed when this miRNA was overexpressed, whereas miR-204 depletion mostly resulted in mild gene expression changes and unaffected cell motility. This could be explained by the relatively low endogenous miR-204 levels due to partial DNA methylation of the *miR*-*204* locus in HaCaT cells (see above), despite being a non-tumorigenic cell line. Since interfering endogenous miR-204 levels in HaCaT cells would likely result in subtle gene expression changes, from then on the analysis of miR-204-mediated changes was carried out by miR-204 overexpression.

### MiR-204 affects the expression of EGF-modulated genes and regulates STAT3 signaling

**Fig. 4:**
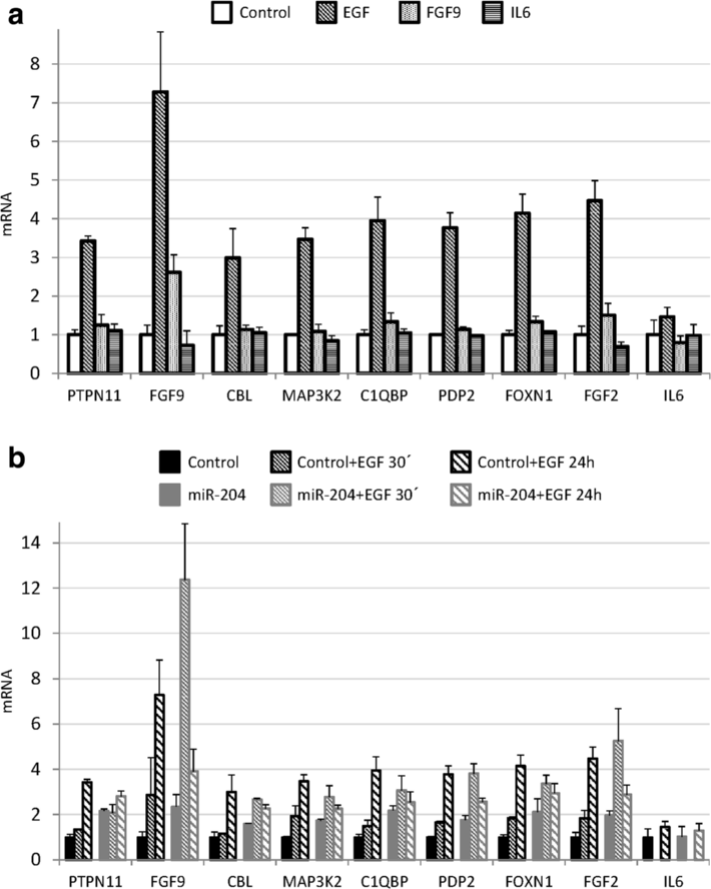
EGF induction of miR-204 modulated genes in HaCaT cells. **a** HaCaT cells were treated with EGF, FGF9 or IL6 (10 ng/ml). Twenty four hours later, total RNA was extracted and mRNA levels of the different transcripts were determined by qRT-PCR. Mean ± SD of three replicate samples from one representative experiment out of two. **b** HaCaT cells were transiently transfected for 24 h with miR-204 mimic or control mimic molecules and treated with EGF (10 ng/ml) for the indicated time points. Mean ± SD of three replicate samples from one representative experiment out of two

The FGF-STAT3 signaling pathway was identified by the IPA software to be modulated by miR-204 expression in HaCaT cells. Therefore we checked whether EGF, IL6 and FGFs, all of them capable of activating MAPK and STAT3 pathways to different extents, modulate the expression of some of the genes differentially expressed in our array. HaCaT treatment with EGF for 24 h resulted in enhanced expression of all the genes tested, but not IL6 gene, that was used as a negative control. In contrast, neither FGF9 nor IL6 were able to modulate the expression of any of the tested genes, exception made for FGF9, which seemed to autoregulate its own expression (Fig. 4a).

Next we tested whether miR-204 overexpression impinged on EGF-induced gene expression. To this end, HaCaT cells transfected with control or miR-204 mimics were stimulated with EGF for 30 min or 24 h. MiR-204 mimic-transfected cells displayed higher basal and induced mRNA levels of the tested genes at short time points. However, mRNA levels of these genes were further increased in control cells at 24 h, whereas the levels of these mRNAs dropped in miR-204 mimic cells (Fig. 4b). These results indicated that miR-204 expression modulates EGF signaling intensity and timing, with an initial activation followed by a downregulation at longer time periods.

**Fig. 5:**
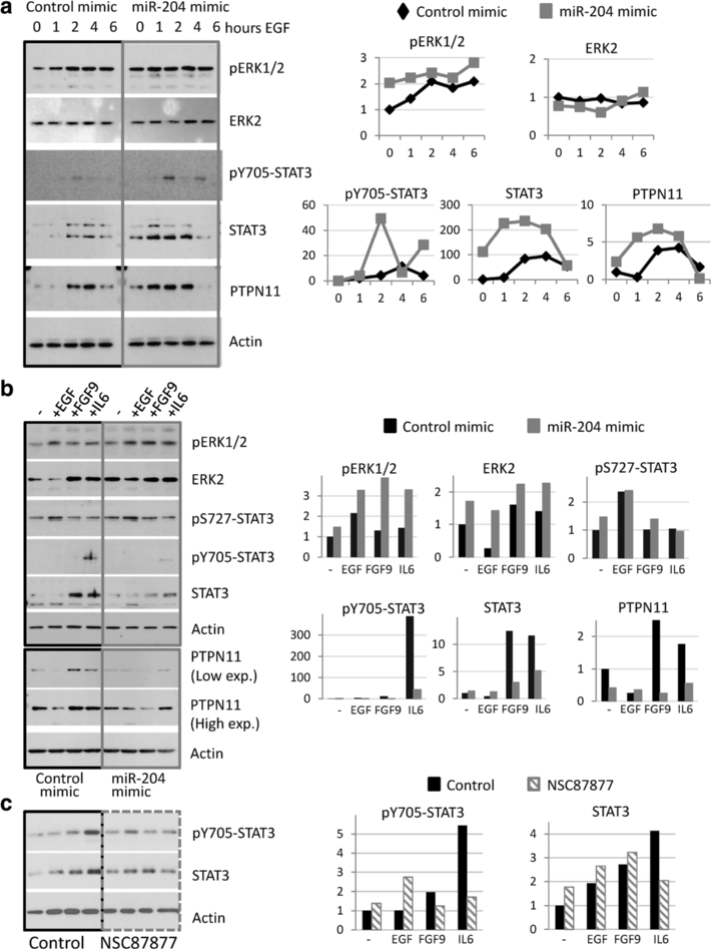
Effects of miR-204 overexpression on MAPK and STAT3 signaling pathways. **a** HaCaT cells were transiently transfected for 24 h with miR-204 or control mimics, and treated with EGF (10 ng/ml) for the indicated time points. Total extracts from these cells were subjected to western blot analysis (*left*). Intensity of individual bands was normalized to Actin signal, as a measure of protein relative abundance in the different samples and referred to control conditions, using Image J densitometry software (*right panels*). **b** Control or miR-204 mimic transfected HaCaT cells were treated with EGF, FGF9 or IL6 (10 ng/ml) for 24 h. Total extracts from these cells were subjected to western blot analysis (*left*). Intensity of individual bands was normalized to Actin signal, as a measure of protein relative abundance in the different samples and referred to control conditions, using Image J densitometry software (*right panels*). **c** HaCaT cells were treated with EGF, FGF9 or IL6 (10 ng/ml) for 24 h, in the absence or in the presence of 50 μM NSC87877, and processed as in **b**

It has been described that EGF stimulation of HaCaT cells induces MAPK activation but fails to phosphorylate STAT3 [48, 49], which is only activated in malignant progression of the keratinocyte towards SCC [50–52]. Therefore, we explored the impact of miR-204 on EGF-mediated regulation of MAPK and STAT3 pathways. HaCaT cells transfected with miR-204 or control mimics were stimulated with EGF for different time periods up to 6 h. ERK2 total levels and ERK1/2 phosphorylation kinetics were similar upon EGF stimulation in both conditions. Of note, pERK1/2 basal levels were higher in miR-204 mimic cells (Fig. 5a). In addition, EGF enhanced STAT3 total levels during the first hours, dropping to basal levels after 6 h of the treatment, being this effect stronger in those cells transfected with miR-204 mimic. Basal pY705-STAT3 levels were undetectable both in control as well as in miR-204-overexpressing cells. EGF treatment induced a biphasic STAT3 phosphorylation at Y705 in miR-204 overexpressing cells in the first hours, whereas pY705-STAT3 was barely detectable in control cells (Fig. 5a).

The miR-204 target PTPN11 can modulate STAT3 [53–55]. Accordingly, the kinetic of PTPN11 expression after the stimulation with the cytokine closely resembled that of total STAT3 and, as observed for STAT3, enhanced PTPN11 expression was stronger in miR-204 mimic cells than control cells at these time points (Fig. 5a).

STAT3 can be activated in a biphasic pattern, with a first wave of activation within 1 h of cytokine treatment and a sustained wave in which STAT3 remains active for many hours [56]. One of the cytokines able to induce sustained STAT3 activation is IL6 [56]. We analysed MAPK and STAT3 pathways after 24 h of treatment with EGF, FGF9 and IL6. At this time point pERK1/2 levels were higher in miR-204 mimic cells stimulated with EGF, FGF9 and IL6 (Fig. 5b). In contrast, control cells displayed enhanced STAT3 expression by FGF9 and IL6 and sustained Y705-STAT3 phosphorylation by IL6 that were not observed in miR-204 overexpressing cells. It has been reported that Y705-STAT3 phosphorylation can be negatively modulated by ERK induced phosphorylation of S727-STAT3 [57]. However, similar levels of S727 phosphorylation could be observed regardless miR-204 expression. PTPN11 levels were higher in untreated cells as well as upon FGF9 and IL6 treatments in control cells when compared to similar conditions in miR-204 overexpressing cells at this time point. All these data indicated that miR-204 induced STAT3-mediated signaling at short time points but resulted in impairment in STAT3 expression and activation triggered by different cytokines signaling through this pathway at longer time points.

PTPN11 has been previously reported to regulate the STAT3 signaling pathway in different cellular contexts. To directly address its role in our experimental conditions, HaCaT cells were stimulated for 24 h with EGF, FGF9 and IL6 in the presence of NSC87877, that specifically inhibits PTPN11 [58], and activated and total STAT3 levels were analysed by western blot. Control cells displayed sustained Y705-STAT3 phosphorylation and enhanced STAT3 expression by IL6 that were not observed in NSC87877 treated cells (Fig. 5c). This result parallels that of the effect of IL6 in miR-204 overexpressing HaCaT cells, supporting the notion that PTPN11 mediates, at least in part, some of the miR-204 regulatory effects on STAT3 signaling pathway.

### Differential subcellular localization of pY705-STAT3 in non-peritumoral and cSCC-adjacent AKs

**Fig. 6:**
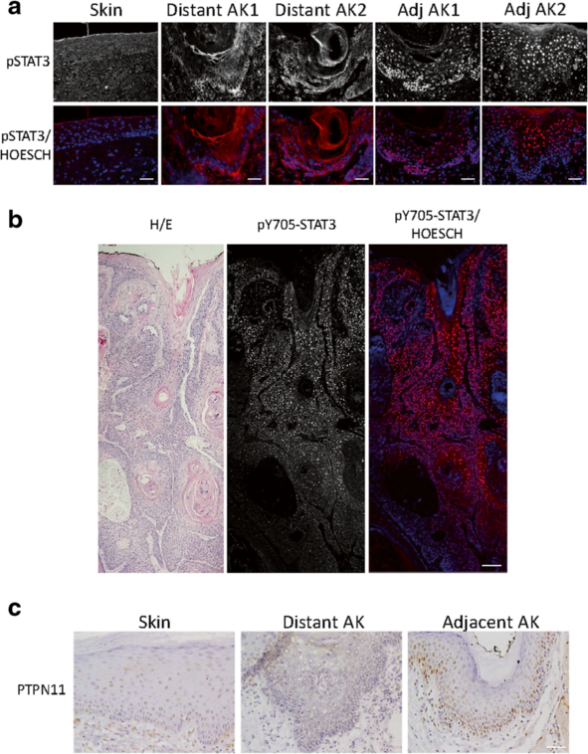
Immunolocalization of pY705-STAT3 in AK and cSCC. **a** pY705-STAT3 signal detected by immunofluorescence in CSE skin, two representative non-peritumoral AK lesions and in two AK lesions adjacent to cSCC. *Scale bar*, 50 μm. **b** Hematoxylin/eosin and pY705-STAT3 staining of cSCC. Observe the intense pY705-STAT3 signal in tumor areas closer to the skin surface whereas the signal is lost in more infiltrative deep regions of the tumor. *Scale bar*, 200 μm. **c** PTPN11 immunodetection in CSE skin, non-peritumoral AK and AK adjacent to cSCC. *Scale bar*, 50 μm

It has been reported that about 80 % of cSCCs display STAT3 activation, localized mainly in the nuclei. Interestingly, AKs also displayed STAT3 activation if the adjacent SCC was positive for STAT3 phosphorylation signal [59, 60]. Since our in vitro data suggested that miR-204 expression in AK would impair long term activation of STAT3 signaling, we investigated pY705-STAT3 localization in 14 non-peritumoral AKs, 11 cSCCs and in their adjacent corresponding AKs. Four of non-peritumoral AKs displayed poor pY705-STAT3 signal and 10 AKs (71 %) displayed pY705-STAT3 signal in the cell membrane and/or cytoplasm, whereas CSE skin was negative (Fig. 6a). In contrast, 9 out of 11 cSCC biopsies and their adjacent AKs (82 %) displayed nuclear pY705-STAT3 signal (Fig. 6a, b), suggesting that STAT3 activation and nuclear localization in AK and cSCC inversely correlate with miR-204 expression. Importantly, nuclear pY705-STAT3 signal in cSCC samples was consistently found in regions closer to the epidermis, whereas deeper and more invasive regions of the tumors were negative for STAT3 activation (Fig. 6b), pointing to an essential role in early stages of the tumorigenesis but not in cSCC progression. PTPN11 was not detected in CSE skins or in distant AKs, whereas cSCC-adjacent AKs displayed nuclear expression (Fig. 6c), suggesting that PTPN11 regulation of STAT3 transcriptional activity takes place in the nucleus.

## Discussion

MiRNAs alterations are involved in the development of several skin cancers [61]. We have identified miR-204, previously found to be down-regulated in head and neck squamous cell carcinoma (HNSCC) [62, 63] and cSCC [25], as the only miRNA to be significantly downregulated in cSCC when compared to AK. MiR-204 is located at 9q21.1-q22-3, a cancer-associated genomic region exhibiting a high frequency of loss of heterozigosity (LOH) in HNSCC, but rarely lost in cSCC. DNA methylation represents the most studied epigenetic regulatory mechanism of modifying gene expression in both coding and non-coding genes such as microRNAs [64–67]. We here report hypermethylation of the miR-204 promoter in AKs and in a high proportion of cSCCs. Since this genomic region also displays STAT3-binding sites, and phosphorylated STAT3 suppresses miR-204 expression in pulmonary arterial hypertension [68], STAT3 activation by alterations in proteins modulating this pathway could account as an alternative mechanism for miR-204 dowregulation in cSCC [69]. Therefore, different DNA-methylation dependent and independent mechanisms could mediate miR-204 repression in cSCC.

Several studies in epithelial cell lines have shown that members of the miR-200 family are repressed during epithelial-to-mesenchymal transition (EMT) [70]. Some in vitro studies with hepatic, renal, ovarian and breast cancer cell lines have shown that loss of miR-204 results in activation of an EMT, leading to cancer cell migration and invasion [71, 72]. We have previously described that EMT is common in metastatic cSCC [73]. Although we have not detected a relationship between miR-204 and EMT, the demonstration of miR-204 silencing in cSCC samples regardless of their biological behaviour makes unlikely the possibility that miR-204 plays a relevant role in this process.

All miRNAs have complex multifunctional and cell context-dependent roles as either repressors or enhancers [18]. Accordingly, we observed a positive short-term induction of the STAT3 pathway by miR-204, whereas in the long-term miR-204 downregulates STAT3 pathway, while favouring MAPK pathway activation. Conversely, low levels of miR-204 in cSCC would favour STAT3 pathway activation, which is also mediated by IL6 [56]. In pulmonary hypertension an auto-regulatory positive feedback has been reported, in which STAT3 activation might perpetuate miR-204 downregulation [74].

The MAPK/ERK pathway is the most important cell survival pathway in non-tumorigenic keratinocytes, and is triggered by EGF. Forced expression of EGFR in immortalized keratinocytes (HaCaT cells) has been associated with enhanced EGFR activation but not with Y705-STAT3 phosphorylation [51]. In contrast, malignant progression of the keratinocyte towards SCC is associated with negative MAPK regulation and EGFR-induced STAT3 activation [50–52], which drives expression of multiple antiapoptotic molecules [50, 52, 75–78]. Accordingly, STAT3 expression was found to be consistently increased in cSCC [59, 60, 79, 80]. Immunofluorescence detection of pY705-STAT3 signal suggests an inverse correlation between the pY705-STAT3 subcelular localization and miR-204 expression in AK and cSCC. Non-peritumoral distant AKs showed phosphorylated STAT3 excluded from the nuclei, whereas AKs adjacent to cSCC displayed nuclear pY705-STAT3 signal when their corresponding tumors were also positive for this marker. Nuclear p705-STAT3 signal in adjacent AKs and cSCC [59, 60] (our own data) suggests that miR-204 already suffers a downregulation in some areas of the AK that will progress to cSCC. On the contrary, pY705-STAT3 signal at the plasma membrane/cytoplasm in some of the non-peritumoral AKs suggests that certain degree of STAT3 activation is already present in AK, but miR-204 expression would inhibit the expression of proteins regulating effective pY705-STAT3 translocation to the nucleus, where these proteins act as transcription factors.

## Conclusions

MicroRNA-204 may act as a “rheostat” that controls the signaling towards the MAPK pathway or the STAT3 pathway. MiR-204 would favour MAPK pathway and make the AK cell insensitive to the induction of the STAT3 pathway despite the presence of procarcinogenic inflammatory mediators. In most cSCCs, miR-204 promoter DNA methylation accounts for miR-204 silencing, which would result in STAT3 activation and translocation to the nucleus, a common feature found in 80 % of cSCCs.

## Abbreviations

cSCC, cutaneous squamous cell carcinoma; AK, actinic keratosis; KIN, keratinocyte intraepidermal neoplasm; CSE skin, chronically sun-exposed skin; HNSCC, head and neck squamous cell carcinoma; EMT, epithelial-to-mesenchymal transition; microRNA, miRNA; IPA, Ingenuity Pathway Analysis

## Additional file

Additional file 1: Clinicopathological data of the patients. (DOCX 106 kb)
