# Supplementary material for: MiR-204 silencing in intraepithelial to invasive cutaneous squamous cell carcinoma progression
Source: Mol Cancer. 2016 Jul 25;15:53. doi: 10.1186/s12943-016-0537-z (PMC4960761; doi:10.1186/s12943-016-0537-z)
Supplement: Additional file 1: — Clinicopathological data of the patients. (DOCX 106 kb) [file 12943_2016_537_MOESM1_ESM.docx]

**Supplementary Methods**

Wound healing assay

HaCaT cells were transiently transfected with miR-204 antagomir, miR-204 mimic or with the corresponding hairpin inhibitor or precursor negative controls (50 nM each) with DHARMAFECT 4. Twenty four hours later, cells were incubated with 10 μg/ml Mitomycin C (Sigma, St. Louis, MO, USA) for 1 h and confluent monolayers were scratched with a plastic pipette tip. Healing was measured at 0 and 24 hours by using Image J software and expressed as a percentage of the area of the scratch remaining unfilled.

Cell cycle analysis

For in vitro BrdU labeling, HaCaT cells were transiently transfected as for wound healing assays, and 24 hours later were pulsed labeled with BrdU for 45 minutes. Then cells were fixed, permeabilized and intracellularly stained with anti-BrdU-FITC using the BrdU Flow Kit (BD Biosciences) and DAPI to gauge the amount of DNA per cell.

| **Gender** | **Age** | **Histology** | **Diameter (cm)** | **Immunosupression** | **Anatomical area** | **Differentiation / KIN** |
| --- | --- | --- | --- | --- | --- | --- |
| **M** | **71** | **cSCC** | **1.8** | **No** | **UK** | **Moderately** |
| **M** | **69** | **cSCC** | **2.1** | **No** | **Forehead** | **Well** |
| **M** | **58** | **cSCC** | **8.5** | **No** | **Leg** | **Well** |
| **M** | **99** | **cSCC** | **1.4** | **No** | **Forearm** | **Moderately** |
| **M** | **73** | **cSCC** | **2.2** | **No** | **Scalp** | **Moderately** |
| **M** | **68** | **cSCC** | **0.6** | **No** | **Temple** | **Moderately** |
| **M** | **88** | **cSCC** | **3.0** | **No** | **Malar** | **Moderately** |
| **M** | **58** | **cSCC** | **1.3** | **No** | **Preauricular** | **Moderately** |
| **F** | **82** | **cSCC** | **1.8** | **No** | **Nose** | **Moderately** |
| **M** | **81** | **cSCC** | **1.6** | **No** | **Scalp** | **Moderately** |
| **M** | **84** | **cSCC** | **2.0** | **No** | **Scalp** | **Moderately** |
| **M** | **82** | **cSCC** | **2.0** | **No** | **Forehead** | **Moderately** |
| **M** | **92** | **cSCC** | **1.0** | **No** | **Temple** | **Moderately** |
| **M** | **81** | **cSCC** | **2.0** | **Yes *** | **Forehead** | **Moderately** |
| **M** | **81** | **cSCC** | **1.5** | **No** | **Scalp** | **Moderately** |
| **M** | **95** | **cSCC** | **6.0** | **No** | **Ear** | **Moderately** |
| **M** | **93** | **cSCC** | **3.0** | **Yes **** | **Arm** | **Moderately** |
| **M** | **79** | **cSCC** | **2.0** | **No** | **Scalp** | **Moderately** |
| **M** | **85** | **cSCC** | **1.3** | **No** | **Malar** | **Moderately** |
| **M** | **94** | **cSCC** | **1.8** | **No** | **Scalp** | **Moderately** |
| **F** | **83** | **AK** | **UK** | **No** | **Malar** | **III** |
| **M** | **79** | **AK** | **UK** | **No** | **Forehead** | **III** |
| **M** | **58** | **AK** | **UK** | **Yes ***** | **Temple** | **III** |
| **F** | **88** | **AK** | **UK** | **No** | **Mandibular area** | **III** |
| **M** | **70** | **AK** | **UK** | **No** | **Forehead** | **III** |
|  | **96** | **CSE** | **NR** | **No** | **Scalp** | **NR** |
|  | **90** | **CSE** | **NR** | **No** | **Malar** | **NR** |
|  | **82** | **CSE** | **NR** | **No** | **Leg** | **NR** |
|  | **53** | **CSE** | **NR** | **No** | **Eyelid** | **NR** |
|  | **78** | **CSE** | **NR** | **No** | **Malar** | **NR** |

**Supplementary Figure S1. Clinicopathological data.** cSCC, Cutaneous invasive squamous cell carcinoma; AK, Actinic keratosis; CSE, Chronically sun-exposed skin. *Methotrexate and Prednisone for Rheumatoid arthritis; **Chronic Lymphatic Leukemia; *** Renal organ recipient. UK, Unknown; NR, Non relevant.

**Supplementary Figure S2.** Primary heat map of genes differentially expressed in HaCaT cells infected with anti-miR-204 cells. In red, sequences validated by qRT-PCR.


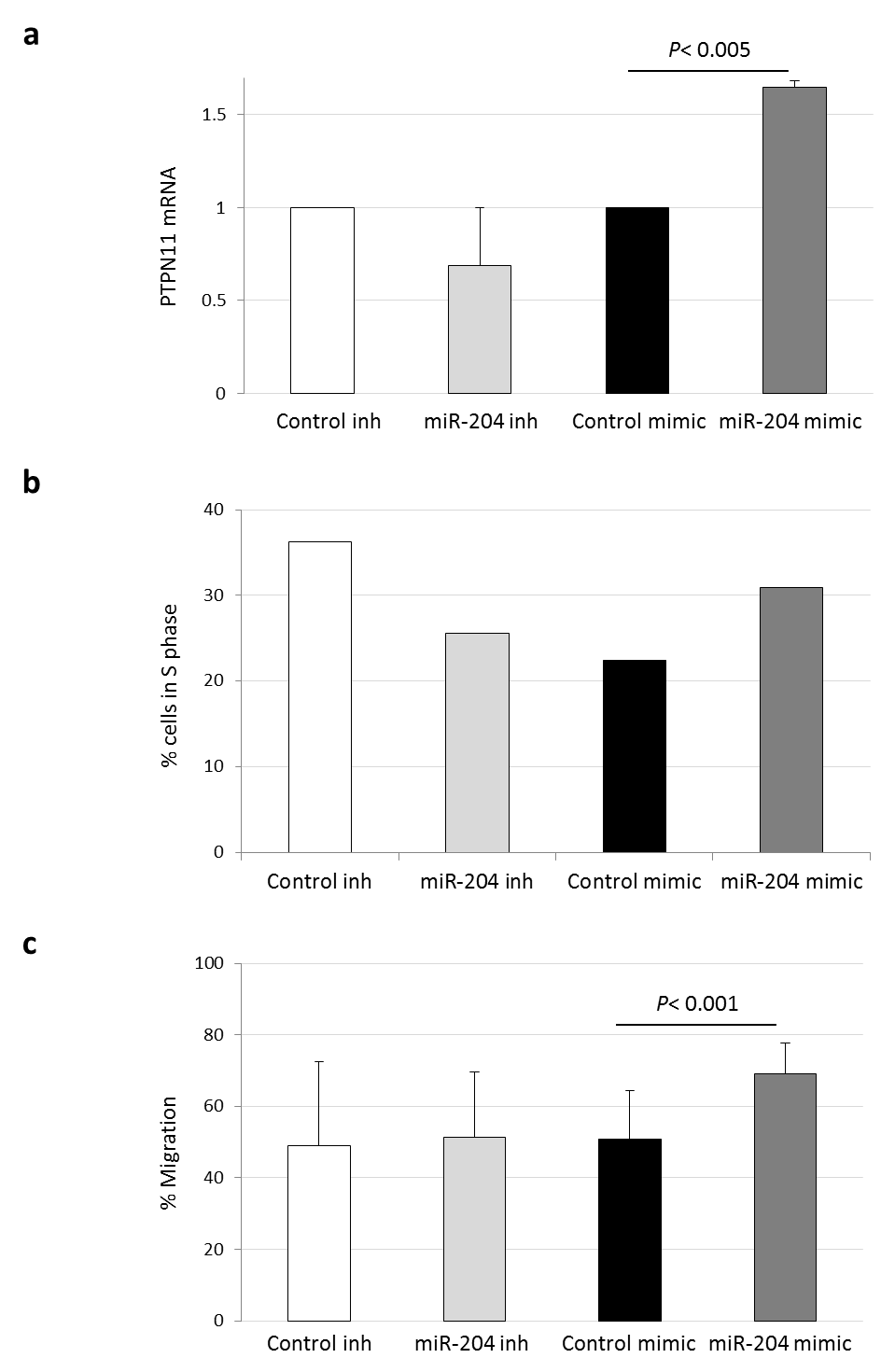


**Supplementary Figure S3.** A. MiR-204 regulation of PTPN11 mRNA levels in HaCaT cells transiently transfected with miR-204 antagomir or mimic along with their controls. Mean ± SD of three replicate samples from two representative experiments. B. BrdU incorporation during S phase into HaCaT cells transfected with miR-204 antagomir or mimic along with their corresponding controls. C. MiR-204 mimic induces HaCaT cell migration, determined by scratch assays. Graph shows data (mean ± SD) from two wound healing assays performed in triplicate.
